# Research on diagnosis method of series arc fault of three-phase load based on SSA-ELM

**DOI:** 10.1038/s41598-021-04605-w

**Published:** 2022-01-12

**Authors:** Bin Li, Shihao Jia

**Affiliations:** Faculty of Electrical and Control Engineering, Liaoning Technical University, Huludao, China

**Keywords:** Engineering, Electrical and electronic engineering

## Abstract

Arc fault in the three-phase load circuit may cause fire, resulting in production interruption and even worse, it will cause casualties. In order to effectively detect the arc fault in the three-phase circuit, series arc fault experiments of three-phase motor load and frequency converter were carried out under different current conditions. Firstly, variational mode decomposition (VMD) was performed for each cycle of A-phase current, and then the VMD energy entropy and sample entropy were calculated. Secondly, the noise-dominated component was removed according to the permutation entropy, then the average value after first-order difference of the half-cycle reconstructed signal was obtained. An arc fault diagnosis model of extreme learning machine (ELM) optimized by sparrow search algorithm (SSA) was established. The feature vectors were divided into training group and test group to train the model and test its fault diagnosis accuracy. Compared with GA-ELM, PSO-ELM, support vector machine (SVM) and SSA-SVM, the experimental results show that the proposed method can identify the series arc fault accurately and more quickly.

## Introduction

There-phase loads such as three-phase motor are widely used in production activities such as resource exploitation. However, the harsh mining conditions and wet environment will accelerate the aging of insulation, meanwhile the mechanical vibration will lead to the loosening of electrical connection, which create conditions for the generation of arc fault. Arc is a kind of gas discharge phenomenon and a kind of plasma, when an arc fault occurs, it is often accompanied by arc light and will emit a large amount of heat, which will further destroy insulation and even cause fire. Therefore, it is of great significance to find a method that can detect the arc fault quickly and effectively.

There are mainly two methods now to detect the arc fault: (1) Detect the arc fault according to the physical characteristics associated with the occurrence of arc, such as local high temperature, arc sound, arc light, etc. However, this method requires the installation of a large number of temperature and optical sensors near the circuit to be detected, it is more suitable for distribution cabinets, switchgear, etc., and its application is limited. (2) Detect the arc fault according to the voltage or current in the time–frequency domain. The detection of arc voltage requires the installation of voltage transformers at many different locations, it is costly and requires huge amounts of data to be observed, which has great limitations. The detection of current only requires the installation of a current transformer on the branch, so researchers mainly detect the arc fault according to the time–frequency characteristics of the current.

Atharparvez et al. compared the power frequency noise and high frequency noise of the current in the normal and fault state, then determined whether the arc fault occurred according to the threshold^1^. Schweitzer Patrick et al. transformed the current signal into two-dimensional feature grayscale images according to the time-domain sequence, and designed a three-layer convolutional neural network with the data set composed of grayscale images to detect the arc fault^2^. Based on the voltage differential protection, Navalpakkam et al. proposed a new method for series arc fault detection of low-voltage system^3^. E. Siegel et al. input the Fourier coefficient, Mel-Frequency Cepstrum data and wavelet characteristics of the current into deep neural networks (DNNs) for the classification of normal state and arc fault^4^. Long et al. adopted the algorithm of Adam optimization and multi-feature fusion neural network to detect the arc fault^5^. Ala et al. used the wavelet coefficient mean difference algorithm and the criterion of four peaks in two cycles combined with the adaptive threshold method to identify the arc fault^6^. Guo et al. obtained the Wigner-Ville distribution value of the signals and thought of them as feature vectors, then input them into the support vector machine optimized by particle swarm optimization algorithm to identify the arc fault^7^. Li et al. found a fast and continuous arc fault detection method based on recursive neural network (RNN) and a classification method based on probability classification result^8^. Qu et al. proposed to use the SOM network optimized by particle swarm optimization to diagnose the arc fault^9^. Park et al. proposed an algorithm comparing current variability in time–frequency domain to realize the arc fault detection^10^. Liu et al. detected arc fault by sparse representation algorithm with adjustable regular order *p* and current amplitude spectrum^11^. Qu et al. input the characteristics in time domain and frequency domain of the current into the learning vector quantization neural network (LVQ-NN) to determine the load type, and detected the arc fault through particle swarm optimization optimized support vector machine (PSO-SVM)^12^. Through chirped Zeta transform of the current signal, Artale et al. performed high-resolution and low-frequency harmonic analysis, and then made appropriate index combination to obtain current feature vectors^13^. Wang et al. transformed the current signal into sparse coefficients by sparse representation fully connected neural network (SRFCNN) and input them into the neural network to diagnose arc fault^14^.

The above methods still have the disadvantages of complex model structure, slow iteration speed and weak anti-interference ability. Therefore, the following method was proposed to diagnose the three-phase series arc fault. Firstly, the current of phase A was decomposed by variational mode decomposition (VMD), and then the VMD energy entropy and sample entropy of the current signal in each cycle were calculated. Secondly, the noise-dominated component was removed according to permutation entropy, and then the current signal was reconstructed. Thirdly, the reconstructed signal was processed by first-order difference, and then the average value of the half-cycle processed current was obtained. Finally, the feature vectors composed of the above three were input into the extreme learning machine optimized by sparrow search algorithm to identify the series arc fault. This method is relatively easy in calculation and high in diagnosis accuracy, can effectively realize the diagnosis of series arc fault of three-phase load.

## Experimental apparatus and phenomena

### Experimental circuit

**Figure 1:**
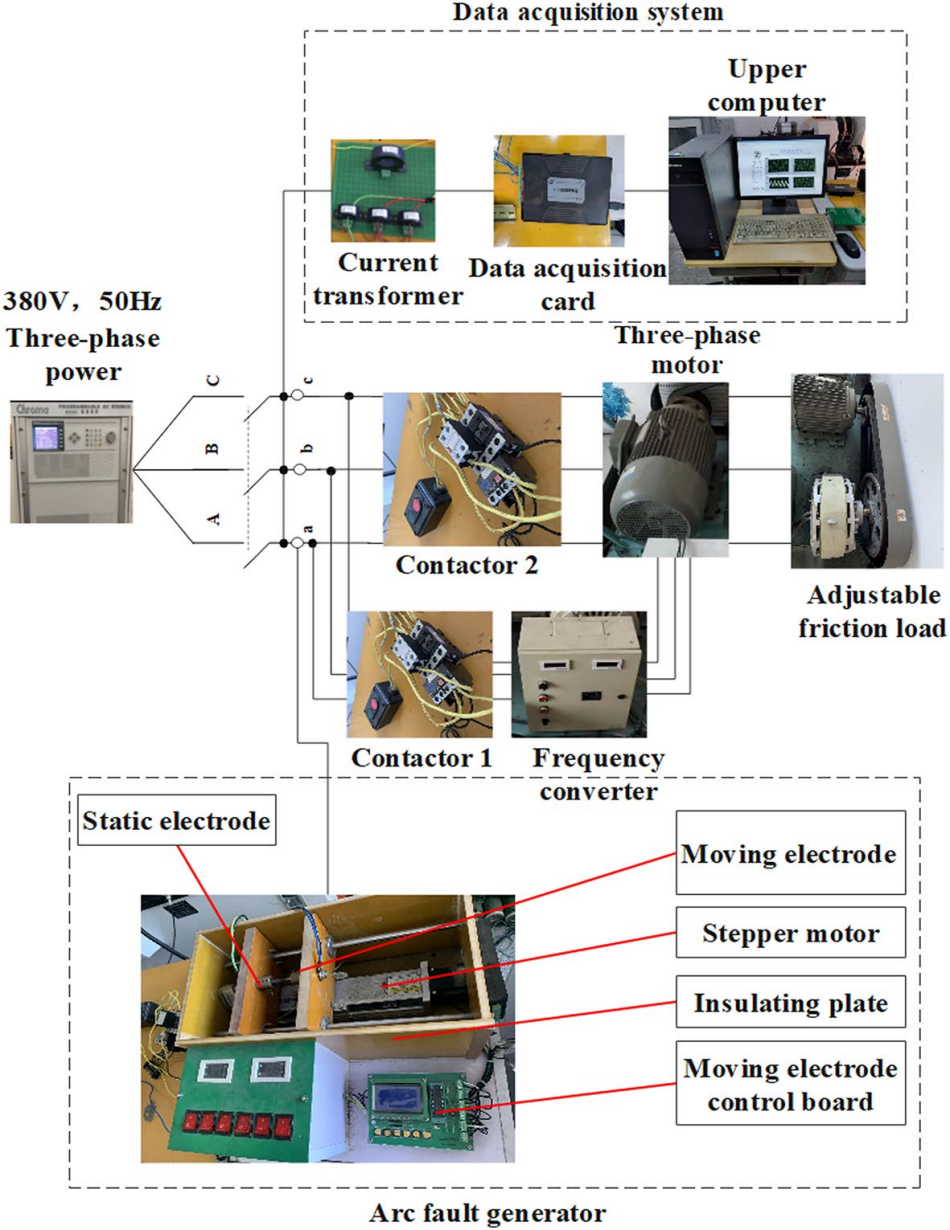
Electrical connection diagram.

The electrical connection diagram of the designed arc fault experimental platform is shown in Fig. 1. The three-phase AC power supply of the experimental platform can output alternating voltage of 380 V and 50 Hz. The type of the three-phase asynchronous motor is Y160M-6-11KW, the type of the frequency converter is VFD110E43A. Based on the experimental platform, series arc fault experiments under different current conditions can be carried out by adjusting the friction load.

The arc fault generator is designed according to the UL1699 standard. The moving electrode is a pointed copper rod with the diameter of 5 mm, and the static electrode is a flat carbon rod with the same diameter. The static electrode is fixed, and the moving electrode, driven by a stepper motor, will gradually approach to the static electrode and not stop until the stable arc is formed. In the fault simulation experiment, the arc fault generator was connected at point a, b and c respectively to simulate the series arc fault of A, B and C phase.

### Experimental scheme

**Figure 2:**
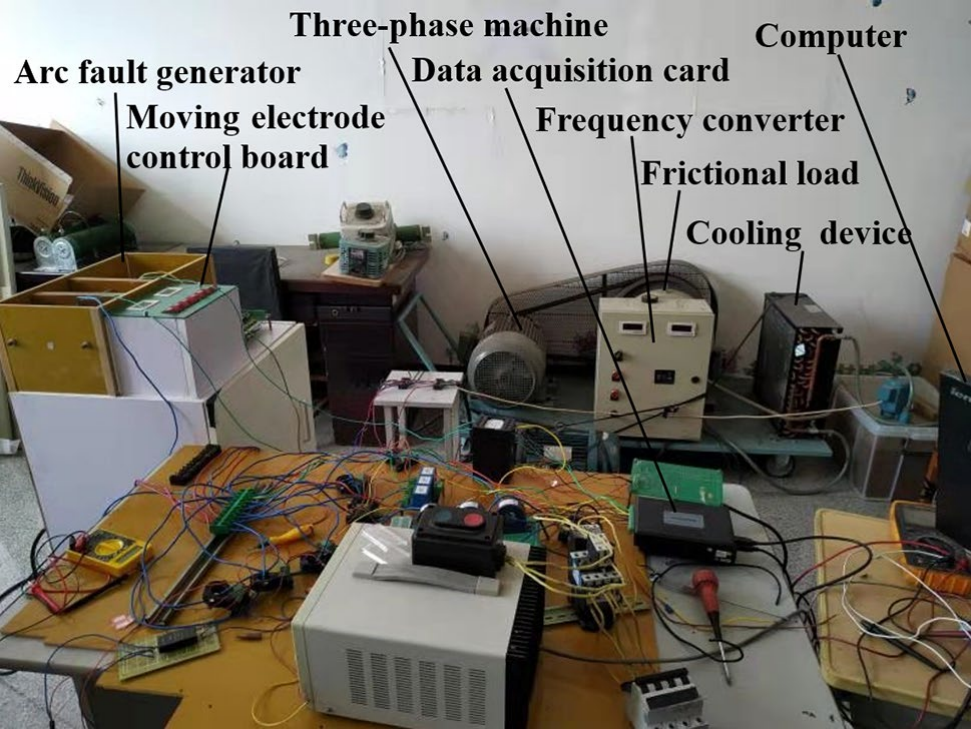
Experimental platform.

The established experimental platform is shown in Fig. 2. The data acquisition system is mainly composed of Hall current transformer, data acquisition card and upper computer. The upper computer is written with LabVIEW software and the control interface can be also seen in Fig. 1. During data acquisition, the Hall current transformer transmits the collected current signal to the upper computer for data recording through the acquisition card.

The experiment in this paper was carried out under the current frequency of 50 Hz, and the current sampling frequency was 100 kHz. So, there were 2000 sampling points in each cycle, and 100,0000 sampling points in total were obtained in each group of the experiment.

In the experiment, by adjusting the friction load, the working current was stabilized at 15A, 20A and a fluctuation state of 15A–20A. 24 experiments in total were carried out and the experimental grouping is shown in Table 1.

**Table 1:** Experimental scheme.

Group	Load	State	Current/A
1–3	Three-phase motor	Normal	15/20/fluctuation
4–6		A-phase fault	
7–9		B-phase fault	
10–12		C-phase fault	
13–15	Three-phase motor and frequency converter	Normal	15/20/fluctuation
16–18		A-phase fault	
19–21		B-phase fault	
22–24		C-phase fault	

### Experimental phenomena

**Figure 3:**
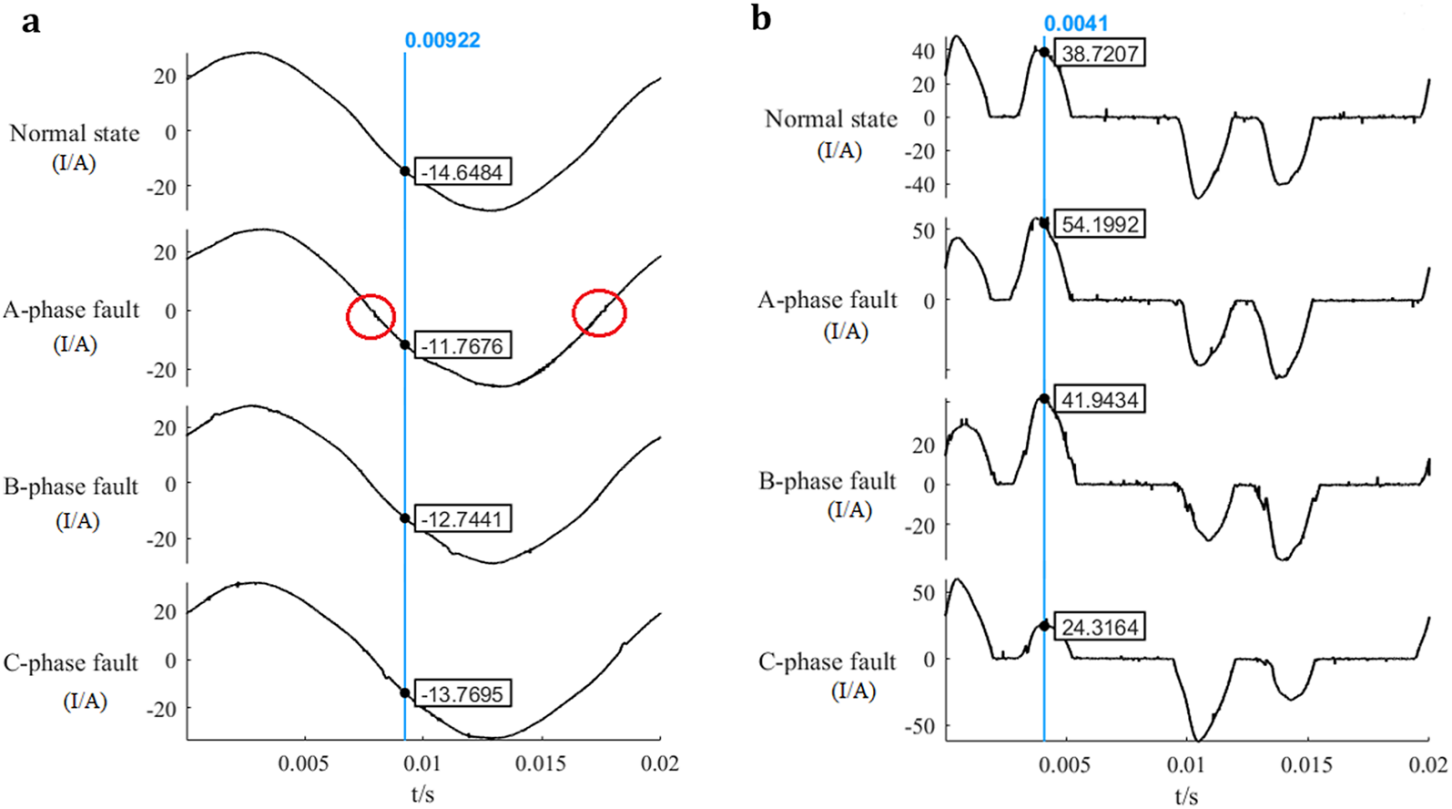
A-phase current waveform of three-phase motor circuit and frequency converter circuit. (**a**) A-phase current waveform of three-phase motor circuit. (**b**) A-phase current waveform of frequency converter circuit.

In the three-phase circuit, the current will flow through A/B phases or A/C phases to form a loop. So, the arc fault of any phase will cause the current change of A-phase, and it can be detected only by analysing the current of phase A. When the working current is 20A, A-phase current waveform of three-phase motor circuit and frequency converter circuit are shown in Fig. 3.

When the load is three-phase motor, there will be burr noise on the current waveform of phase A when the arc fault occurs in any phase. There will be zero-break phenomenon at zero crossing point when the arc fault occurs in phase A, while there will be no zero-break phenomenon when the arc fault occurs in other phases.

When running with frequency converter, which is a nonlinear load, there will be double peaks in the current waveform. When arc fault occurs, the burr noise on the current waveform will increase, the maximum value of the two peaks and the height difference between the two will change.

There are above differences of the current between normal and fault state, we can carry out state diagnosis according to these features.

## Data analysis

### Variational mode decomposition

VMD can decompose the original signal by constructing and solving the constrained variational problem. It can decompose the multi-component signal into several single-component and amplitude-frequency modulated signals, namely the intrinsic mode function (IMF)^15,16^. After removing the noise-dominated component, the remaining components can be reconstructed to achieve the effect of noise reduction. VMD can be represented by the following constrained optimization problems.

1 $$ \begin{array}{*{20}l}    {min}  \\    {\left\{ {u_{k} } \right\},\{ \omega _{k} \} }  \\   \end{array}  = \left\{ {\sum\limits_{1}^{k} {\left\| {\partial _{t} \left[ {\left( {{{\updelta}}\left( {\text{t}} \right) + \frac{{\text{j}}}{{\pi {\text{t}}}}} \right)*{\text{u}}_{k} ({\text{t}})} \right]e^{{ - j\omega t}} } \right\|_{2}^{2} } } \right\}{\text{s}}.{\text{t}}.\sum\limits_{1}^{k} {u_{k} }  = f_{{signal}}  $$

As is shown in Eq. (2), when the quadratic penalty function term and the Lagrange multiplier term are added to Eq. (1), it will be an unconstrained optimization problem^17^.

2 $$ L\left( {\left\{ {u_{k} } \right\},\left\{ {\omega _{k} } \right\},{{\uplambda}}} \right) = \alpha \sum\limits_{1}^{k} {\left\| {\partial _{t} \left[ {\left( {{{\updelta}}\left( {\text{t}} \right) + \frac{{\text{j}}}{{\pi {\text{t}}}}} \right)*{\text{u}}_{k} ({\text{t}})} \right]e^{{ - j\omega t}} } \right\|_{2}^{2} }  + \left\| {f\left( t \right) - \sum\limits_{1}^{k} {{\text{u}}_{k} } ({\text{t}})} \right\|_{2}^{2}  + \left\langle {{{\uplambda}}\left( {\text{t}} \right),{\text{f}}\left( {\text{t}} \right) - \sum\limits_{1}^{k} {{\text{u}}_{k} } ({\text{t}})} \right\rangle  $$

Here ${u}_{k}$ represents the IMF component, ${\omega }_{k}$ represents the central frequency of each IMF component, and ${f}_{signal}$ is the original signal.

The specific process of VMD is as follows:

1. Set the initial value $\left\{{{u}_{k}}^{1}\right\}$, $\left\{{{\omega }_{k}}^{1}\right\}$, ${\lambda }^{1}$, *n* = 0;
2. Start the cycle, *n* = *n* + 1;
3. Set the number of decompositions *k*, then update ${u}_{k}$, ${\omega }_{k}$ and $\lambda$ according to the Eq. (2);
4. Set the precision, stop iteration if the following equation is satisfied, otherwise turn (2) to continue the cycle.
  3 $$\sum_{1}^{k}\frac{{\Vert {{u}_{k}}^{n+1}-{{u}_{k}}^{n}\Vert }_{2}^{2}}{{\Vert {{u}_{k}}^{n}\Vert }_{2}^{2}}<\varepsilon $$

In this paper, four-layer variational mode decomposition was used to process the current signal, so that the fault features could be extracted accurately and the running speed could be guaranteed. VMD parameters were set as follows: penalty factor $alpha=200$, noise tolerance $nt=0$, decomposition layer $k=4$, tolerance of convergence criterion $tc=1e-7$.

**Figure 4:**
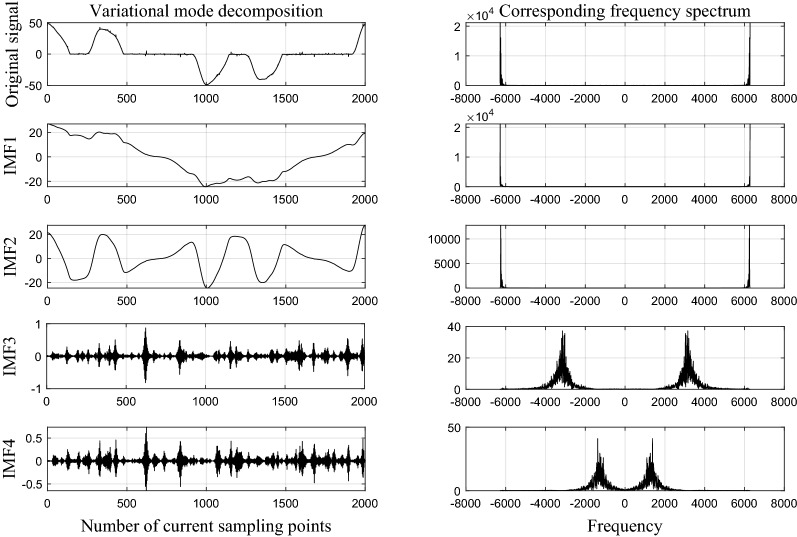
The result of VMD in normal state.

**Figure 5:**
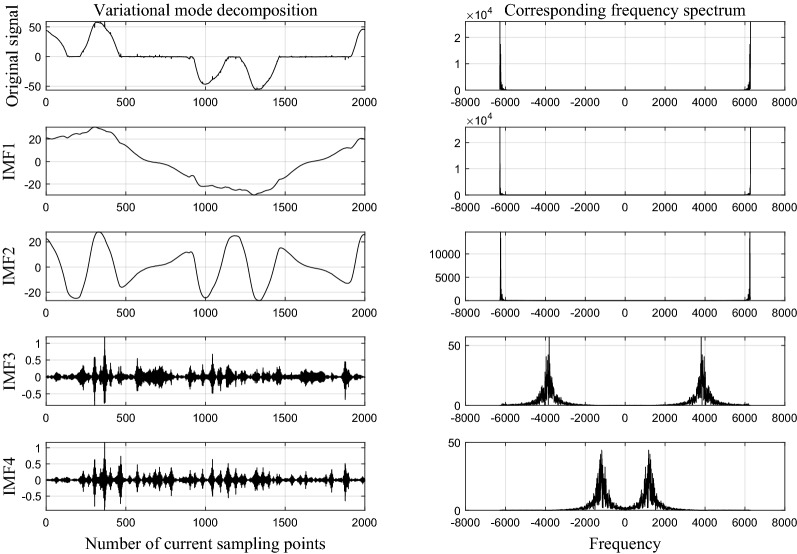
The result of VMD in fault state.

Taking the frequency converter running at 20A as an example, Figs. 4 and 5 show VMD results under normal and fault state respectively. The amplitude of each IMF component increases in the frequency domain when the arc fault occurs, indicating that the occurrence of arc fault makes the time series of current signal complicated.

### Signal reconstruction

Permutation entropy can be used to describe the degree of noise in a signal^18^. The smaller the entropy value is, the more continuous and regular the time series is. The larger the entropy value is, the more irregular and random the time series is^19^.

The permutation entropy of each IMF component under the experimental conditions of 20A current and arc fault in phase A is shown in Table 2.

**Table 2:** Permutation entropy of each IMF component.

Load type	Component	Permutation entropy	Component	Permutation entropy
Three-phase motor	IMF1	0.7999	IMF3	0.9750
	IMF2	0.9760	IMF4	0.9674
Frequency converter	IMF1	0.6519	IMF3	0.9261
	IMF2	0.7982	IMF4	0.9630

During signal reconstruction, the maximum permutation entropy value of IMF components is taken as the threshold value, that is, when the load is three-phase motor, the threshold value is 0.9760, and when running with frequency converter, the threshold value is 0.9630. Components with permutation entropy greater than this value are filtered out and the remaining components are reconstructed. In this way, the useless interference noise is removed from the reconstructed signal, while burr noise caused by the arc fault is retained.

**Figure 6:**
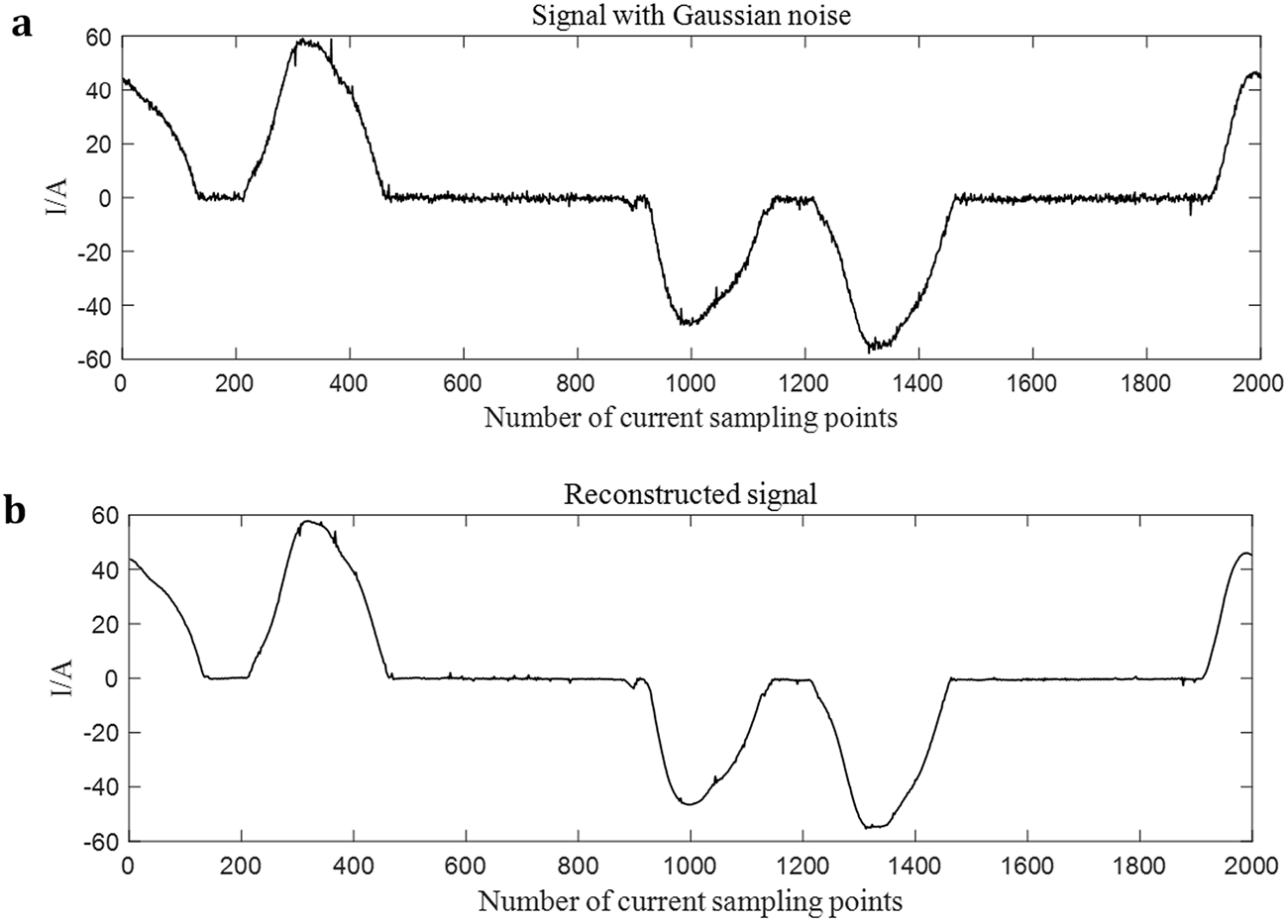
Signal with Gaussian noise and Reconstructed signal. (**a**) signal with Gaussian noise. (**b**) reconstructed signal.

The current signal in the experiment is ideal and there is no interference noise, but there may be noise in the practical application of three-phase load. To verify the denoising effect of VMD, Gaussian noise with a signal-to-noise ratio of 30 was artificially added to the current signal and then the signal was reconstructed according to the above method. The signal with Gaussian noise and the reconstructed signal are shown in Fig. 6.

### Calculation of input feature vectors

#### VMD energy entropy

VMD energy entropy can be used to describe the complexity and uncertainty of current signal. When arc fault occurs, the frequency of each IMF component shows randomness, and the VMD energy entropy increases significantly^20,21^.

Suppose $E(i)$ is the energy of current signal at the decomposition level $i$, the total energy $E=\sum_{i=1}^{4}E(i)$, so ${p}_{i}=\frac{E(i)}{E}$ and $\sum_{i=1}^{4}{p}_{i}=1$. The VMD energy entropy can be defined as:

4 $${H}_{EE}=-\sum_{i=1}^{4}{p}_{i}\mathrm{lg}{p}_{i}$$

**Figure 7:**
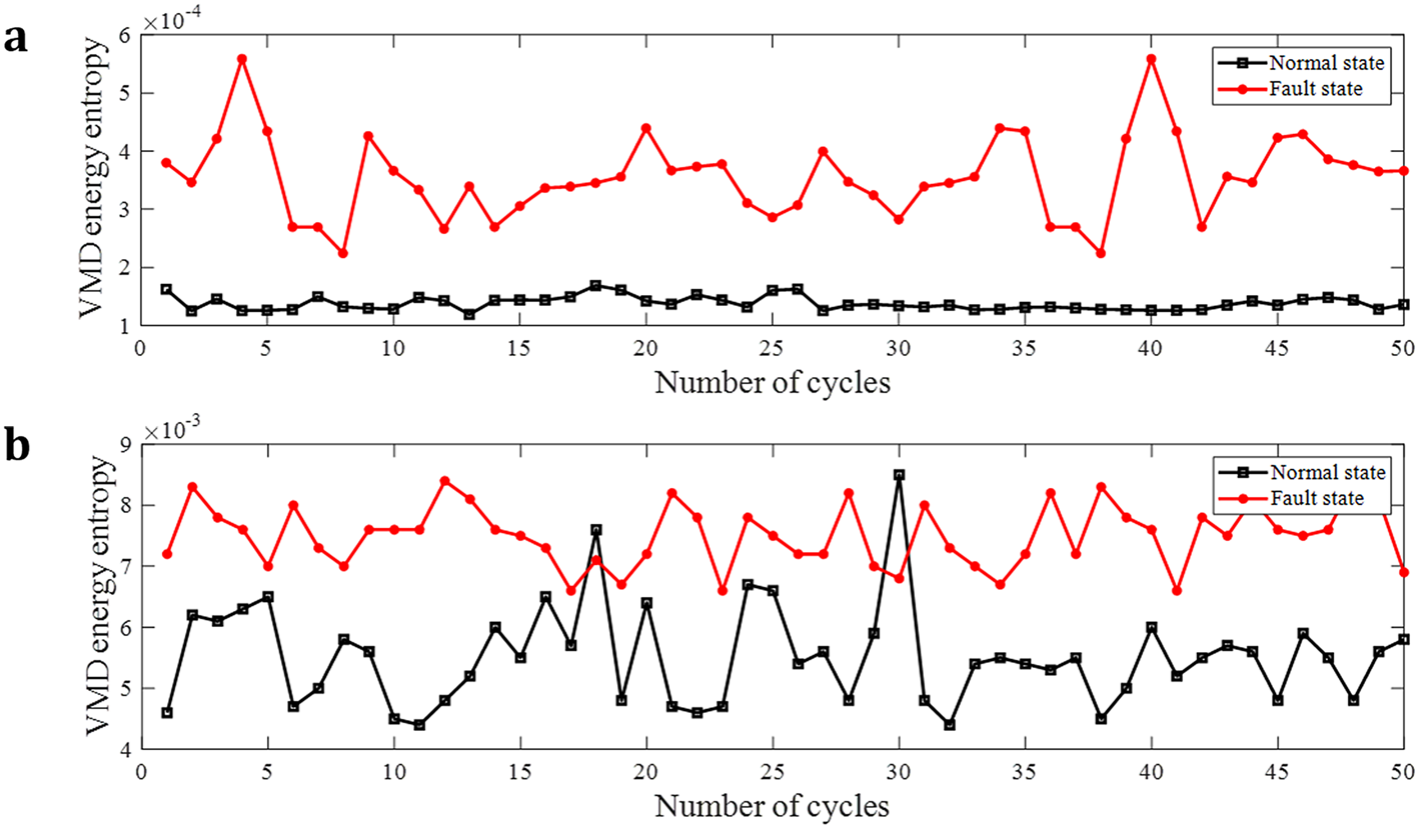
VMD energy entropy of current in three-phase motor circuit and frequency converter circuit. (**a**) VMD energy entropy of current in three-phase motor circuit. (**b**) VMD energy entropy of current in frequency converter circuit.

VMD energy entropy for 50 cycles at 20A are shown in Fig. 7. When the three-phase motor is running, the curves of normal state and fault state are clearly distinguished and there is no overlap, but when running with frequency converter, the curves sometimes cross. So simply using VMD energy entropy is not enough to distinguish normal and fault states.

#### Sample entropy

Sample entropy is the improvement of approximate entropy, which has strong anti-interference ability and is not limited by data length^22^. When arc fault occurs, the current time series will be complicated, resulting in an increase in sample entropy.

The calculation steps of sample entropy are as follows:

1. Decompose the signal with *N* sampling points in one period into multiple vectors of dimension *d*: ${I}_{d}\left(1\right),{I}_{d}(2),\dots ,{I}_{d}(N-d+1)$
2. Assume that the distance between ${I}_{d}(m)$ and ${I}_{d}(n)$ is *l*, then
  5 $$l=\underset{k=\mathrm{0,1},\dots ,d-1}{\mathrm{max}}\left(\left|{I}_{d}\left(m+k\right)-{I}_{d}\left(n+k\right)\right|\right)$$
3. Suppose ${I}_{d}(m)$ is known, calculate the number of vectors ${A}_{m}$, whose distance between ${I}_{d}(m)$ and ${I}_{d}(n)$ is less than *r*, then
  6 $${A}_{m}^{d}\left(r\right)=\frac{1}{N-d-1}{A}_{m}$$
  7 $${A}^{d}\left(r\right)=\frac{1}{N-d}\sum_{m=1}^{N-d}{A}_{m}^{d}\left(r\right)$$
4. Let $k=d+1$, repeat step (3), then
  8 $${B}^{k}\left(r\right)=\frac{1}{N-k}\sum_{m=1}^{N-k}{B}_{m}^{k}(r)$$
  9 $${H}_{SE}=-\mathrm{ln}\frac{{B}^{k}\left(r\right)}{{A}^{d}\left(r\right)}$$

**Figure 8:**
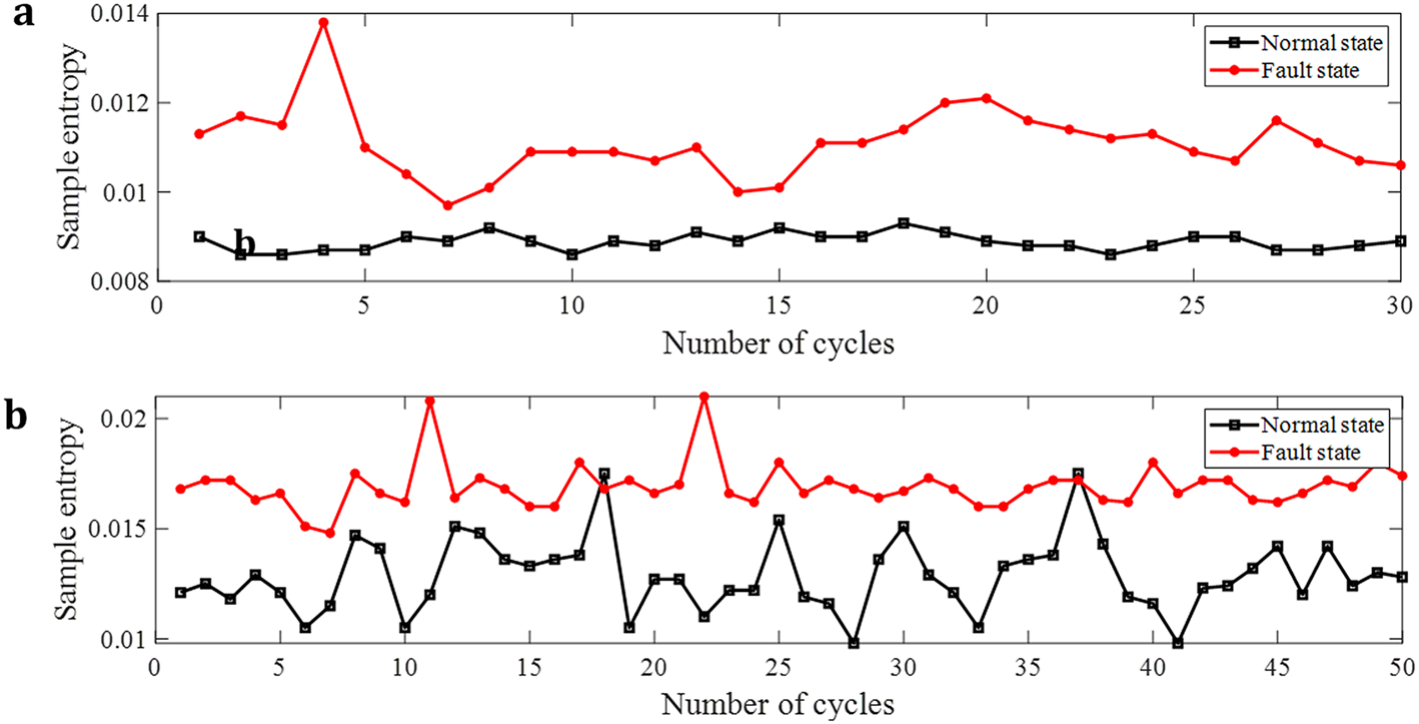
Sample entropy of current in three-phase motor circuit and frequency converter circuit. (**a**) Sample entropy of current in three-phase motor circuit. (**b**) Sample entropy of current in frequency converter circuit.

Sample entropy for 50 cycles at 20A are shown in Fig. 8. When the frequency converter is running, the sample entropy curves of normal and fault states sometimes cross.

#### Average value after first-order difference

When the load is three-phase motor, the occurrence of arc fault may lead to zero-break phenomenon in current waveform. When running with frequency converter, the maximum value of the two peaks and the height difference between the two change because of the arc fault. In addition, the arc fault causes burr noise in current. These differences between normal and fault state can be amplified by the average value after first-order difference $\overline{X }$.

#### Combination of features

It is found in the experiment that the curves of VMD energy entropy and sample entropy under normal or fault conditions will occasionally cross, which makes machine learning difficult, so the use of any single feature cannot meet the accuracy requirements of fault diagnosis. Therefore, in this paper, VMD energy entropy, sample entropy of each cycle and the average value after first-order difference of half cycle were used to form the three-dimensional feature vector $E=[{H}_{EE},{H}_{SE},\overline{X }]$. A total of 500 groups of positive and negative samples were used as the input of the model.

## Extreme learning machine optimized by sparrow search algorithm

### Sparrow search algorithm

Sparrow search algorithm (SSA) is a new swarm intelligence optimization algorithm, which is proposed in 2020, under the inspiration of the sparrow's foraging and anti-predation behaviour^23^. Compared with other swarm optimization algorithms such as genetic algorithm (GA), particle swarm optimization (PSO), and grey wolf optimization (GWO), SSA has the advantages of less adjustable parameters and faster convergence speed^24–26^.

In the process of foraging, the sparrow population can be abstracted into two different types: the discoverer and the participant. The discoverer has high energy reserves and high fitness, and is responsible for finding food for other sparrows in the population and guiding them to move to areas with food. The participant cannot actively search for food and must follow the discoverer.

#### Mathematical model of the discoverer

The identity of the discoverer and the participant can convert into each other. In each iteration, the position of the discoverer is updated according to the following equation:

10 $$ X_{{ij}}^{{t + 1}}  = \left\{ {\begin{array}{*{20}l}    {X_{{ij}}^{t}  \cdot exp\left( { - \frac{i}{{\alpha  \cdot G}}} \right),} \hfill & {R_{2}  < ST} \hfill  \\    {X_{{ij}}^{t}  + Q \cdot L,} \hfill & {R_{2}  \ge ST} \hfill  \\   \end{array} } \right. $$

where *t* is the iteration times,${X}_{ij}$ represents the location information of the *i*th sparrow in the *j*th dimension. *G* represents the maximum iteration number. $\alpha$ is a random number between 0 and 1, ${R}_{2}\in [\mathrm{0,1}]$ denotes the warning value, and $ST\in [\mathrm{0.5,1}]$ denotes the safety value. *Q* is a random number which obeys the normal distribution, *L* is a unit matrix of one row and *d* columns.

${R}_{2}<ST$ indicates that the discoverer has not found danger, ${R}_{2}>ST$ indicates that the discoverer has perceived danger of the predator. When danger occurs, the discover needs to send signal to participants, then move to a safer place.

#### Mathematical model of the participant

The position of the participant is updated according to the following equation:

11 $$ X_{{ij}}^{{t + 1}}  = \left\{ {\begin{array}{*{20}l}    {Q \cdot exp\left( {\frac{{X_{{worst}}^{t}  - X_{{ij}}^{t} }}{{\alpha  \cdot G}}} \right),} \hfill & {i > \frac{n}{2}} \hfill  \\    {X_{p}^{{t + 1}}  + \left| {X_{{ij}}^{t}  - X_{p}^{{t + 1}} } \right| \cdot A^{ + }  \cdot L,} \hfill & {otherwise} \hfill  \\   \end{array} } \right. $$

where ${X}_{p}$ represents the best position of the present discoverer, and ${X}_{worst}$ represents the present worst position in the overall situation. *A* is a matrix with one row and *d* columns, and each element of the matrix is randomly assigned a value of 1 or -1, ${A}^{+}={A}^{T}{({AA}^{T})}^{-1}$. $i>\frac{n}{2}$ indicates that the *i*th participant does not receive food and needs to fly somewhere else to find food.

#### Mathematical model of the anti-predation behavior

When the sparrows find danger, they will update their position according to the following equation:

12 $${X}_{ij}^{t+1}=\left\{\begin{array}{c}{X}_{best}^{t}+\beta \cdot \left|{X}_{ij}^{t}-{X}_{best}^{t}\right|, {f}_{i}>{f}_{g}\\ {X}_{ij}^{t}+K\cdot \left(\frac{\left|{X}_{ij}^{t}-{X}_{worst}^{t}\right|}{\left({f}_{i}-{f}_{w}\right)+\varepsilon }\right), {f}_{i}={f}_{g}\end{array}\right.$$

where ${X}_{best}$ represents the global optimal value under present situation. $\beta$ is the step size control factor, a random number subject to normal distribution. $K\in [-\mathrm{1,1}]$ is a random number, ${f}_{g}$ and ${f}_{w}$ are the global best and worst fitness values respectively, $\varepsilon$ is a constant added to prevent the denominator from being 0.

### Extreme learning machine

**Figure 9:**
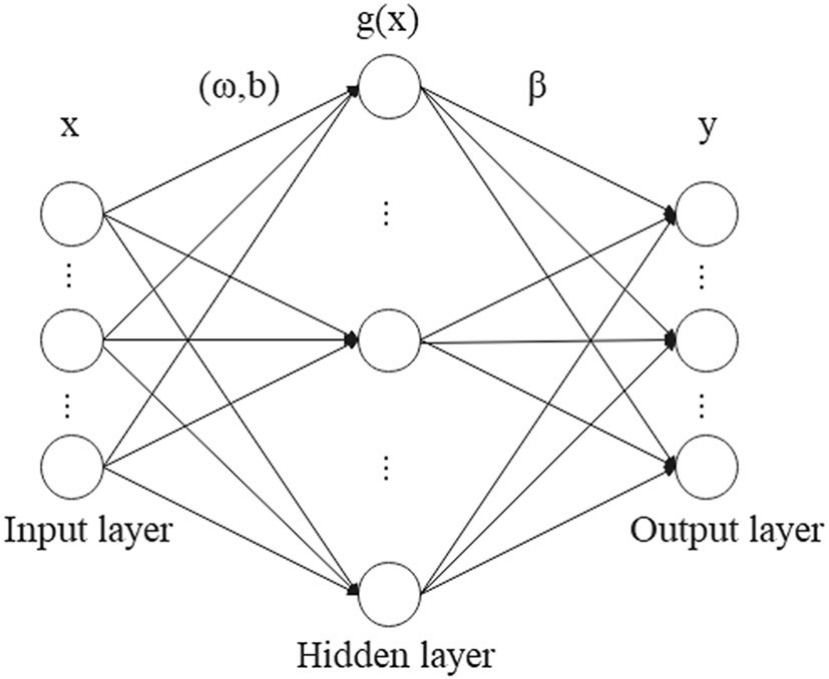
ELM model.

Extreme learning machine (ELM) is a three-layer feedforward neural network and there is only one hidden layer inside it^27,28^. In ELM, the hidden layer biases and connection weights between each layer are automatically generated, only the number of neurons in the hidden layer needs to be set manually. The simple structure makes ELM easy to build and enables ELM to have a faster learning speed than the traditional neural network^29,30^. The structure diagram of ELM is shown in Fig. 9.

In ELM, the number of the input neurons *n* is equal to the dimension of the input feature vectors to be classified, so in this research $n=3$. The number of neurons in the output layer *m* is equal to the number of fault types, so in this research $m=2$. There are a total of *L* neurons in the hidden layer, so the ELM model with *T* training samples can be expressed by the following equation:

13 $${Y}_{T}=\sum_{i=1}^{L}{\beta }_{i}g \left({\omega }_{i}{x}_{j}+{b}_{i}\right) , j=\mathrm{1,2},\dots ,T$$

where ${\beta }_{i}$ represents the connection weight between the neuron in the *i*th hidden layer and the output layer, $g$(x) is the activation function, ${\omega }_{i}$ represents the connection weight between the neuron in the *i*th input layer and the hidden layer, ${x}_{j}$ represents the input vector, and ${b}_{i}$ represents the threshold of the neuron in the *i*th hidden layer.

### Optimization process of SSA-ELM

SSA is used to optimize the number of neurons in the hidden layer of ELM to improve its ability of arc fault diagnosis, the specific steps are as follows:

1. Input the collected and classified feature vectors into ELM. Select some groups as training set to train the model, and the rest as the test set to verify the fault diagnosis accuracy of the model.
2. Set the maximum number of iterations *N*, the size of sparrow population *n*, security threshold *ST*, the ratio of the discoverer to the population *PD*, and the ratio of the number of sparrows aware of danger to the population *SD*.
3. Set the optimization range and initial number of neurons in the hidden layer of ELM, then train the model with the minimum error rate as the goal.
4. Find the optimal number of neurons *L* through sparrow search algorithm. When the optimal value is obtained, output and update it to the established ELM model. Then, using the fault diagnosis model to identify the arc fault.

## Arc fault diagnosis

### Comparison of fault diagnosis effect between SSA-ELM and ELM

In reality, the probability of the occurrence of arc fault is relatively small. In order to make the model more practical, according to the ratio of 4:1, a total of 500 groups of feature vector $E=[{H}_{EE},{H}_{SE},\overline{X }]$ under normal and fault conditions were taken as the input.

400 out of 500 groups were randomly selected as the training group and the remaining 100 as the test group. ELM output to 1 represented normal state and output to 2 represented fault state. The initial number of neurons in the hidden layer was 50, and the optimization range was [1,1000]. The parameters of SSA were set as follows: the size of sparrow population *n* was 20, the maximum number of iterations *N* was 50, the safety threshold *ST* was 0.8, the ratio of discoverer to population size *PD* was 20%, the ratio of the number of sparrows aware of danger to population *SD* was 10%.

**Figure 10:**
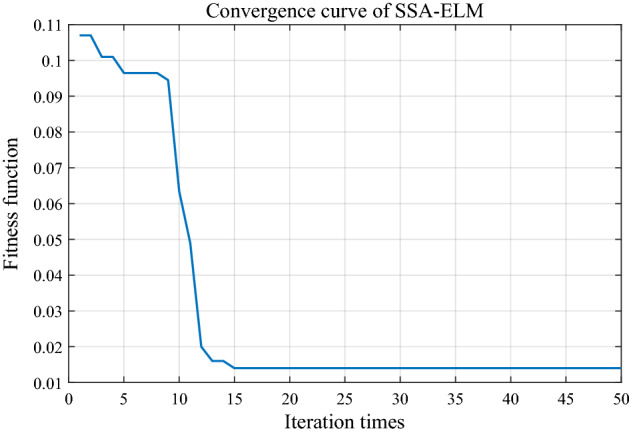
Convergence curve of SSA-ELM.

Taking the error rate as the fitness function and the minimum error rate as the goal, SSA was used to find the best value of the number of neurons in the hidden layer. As shown in Fig. 10, in an experiment the optimal solution is obtained at the 15th iteration. The results show that SSA can iterate to the optimal solution in a very short time.

**Figure 11:**
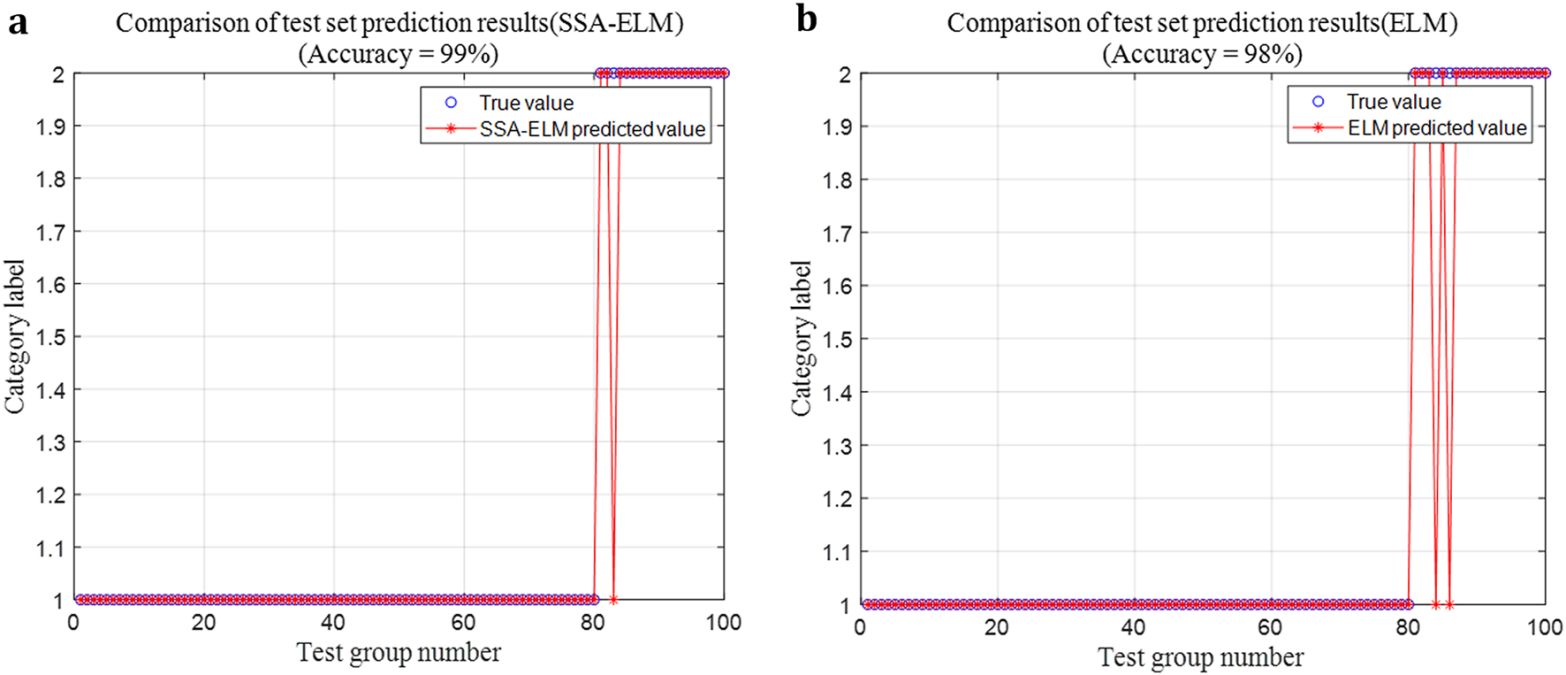
Fault diagnosis accuracy of SSA-ELM and ELM. (**a**) Fault diagnosis accuracy of SSA-ELM. (**b**) Fault diagnosis accuracy of ELM.

Experiments were carried out under the conditions of 15A, 20A and fluctuating working current, the experimental result of SSA-ELM under the condition of 20A current is shown in Fig. 11a. When the number of hidden layer neurons is set at 100 according to experience, the experimental result of ELM is shown in Fig. 11b.

### Comparison of optimization effect between SSA and other methods

**Figure 12:**
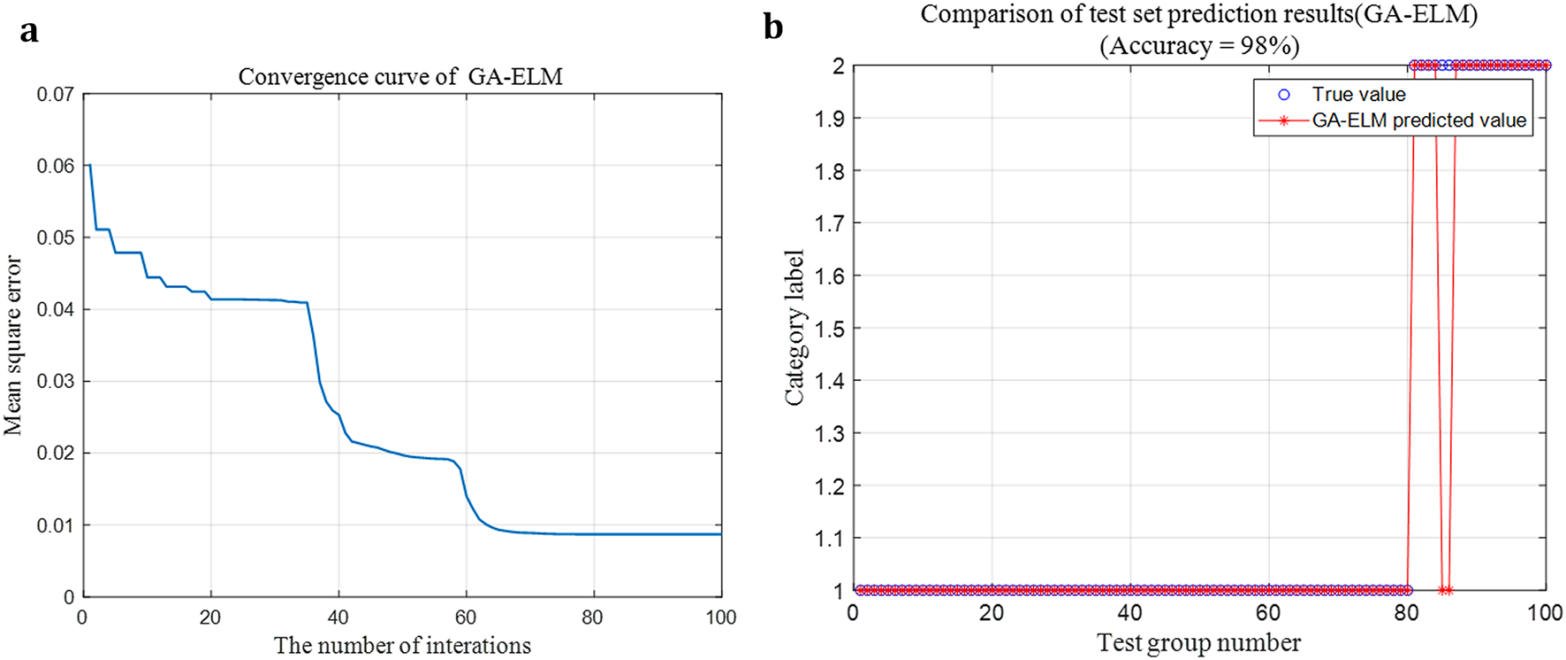
Fault diagnosis effect of GA-ELM. (**a**) Convergence curve of GA-ELM. (**b**) Fault diagnosis accuracy of GA-ELM.

In order to verify the superior optimization effect of SSA, the genetic algorithm (GA) and particle swarm optimization (PSO) were used to optimize the number of hidden layer neurons of ELM. Parameters of the genetic algorithm were set as follows: the maximum number of iterations was 100, the population size was 20, the probability of crossover was 0.7, and the probability of mutation was 0.01. The GA-ELM model was used for arc fault diagnosis, and the results are shown in Fig. 12.

**Figure 13:**
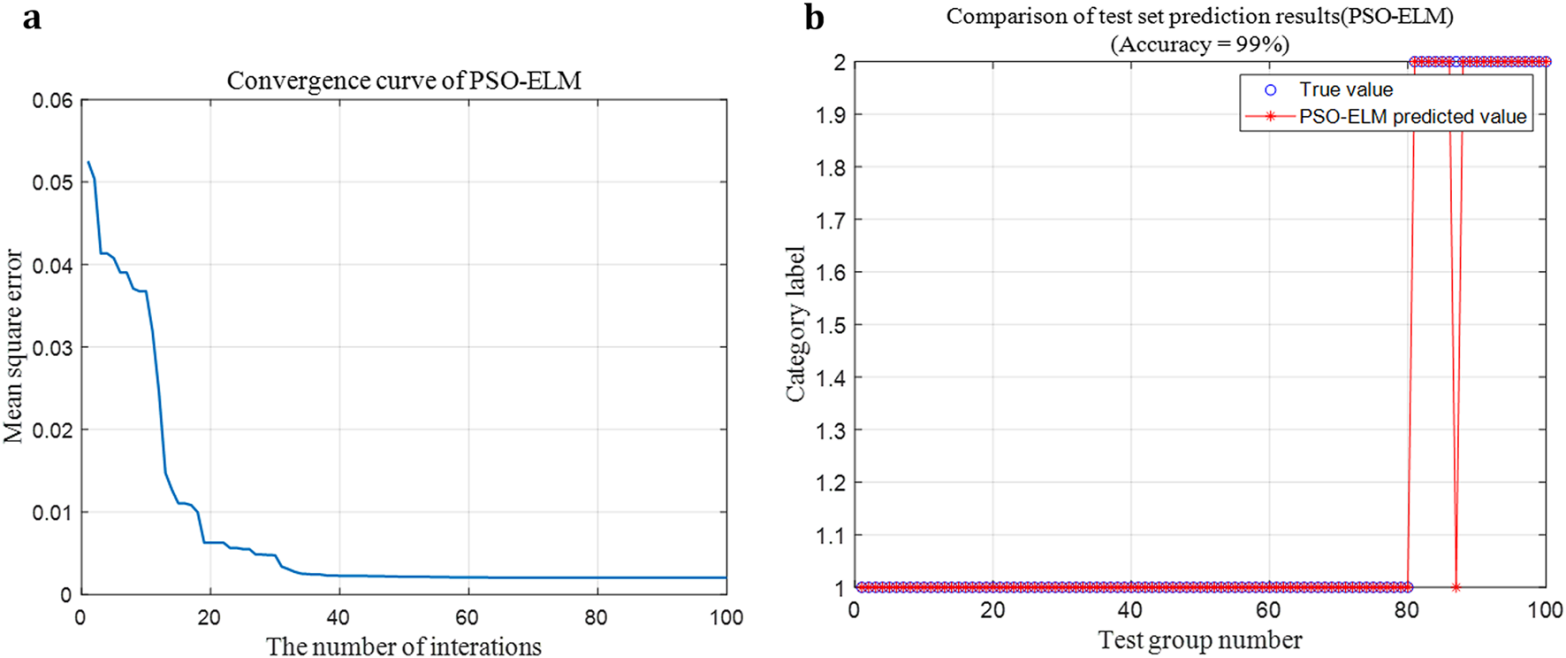
Fault diagnosis effect of PSO-ELM. (**a**) Convergence curve of PSO-ELM. (**b**) Fault diagnosis accuracy of PSO-ELM.

Parameters of the particle swarm optimization were set as follows: the dimension was 3, the acceleration constant was 2, the size of particle swarm was 20, and the maximum number of iterations was 100. The PSO-ELM model was used for arc fault diagnosis, and the results are shown in Fig. 13.

By comparing Figs. 11, 12, 13, it can be found that SSA has the fastest iteration speed, followed by PSO, and GA has the slowest iteration speed. Therefore, SSA-ELM model is more suitable for the rapidity of arc fault diagnosis.

### The effect of SSA on SVM parameter optimization

**Figure 14:**
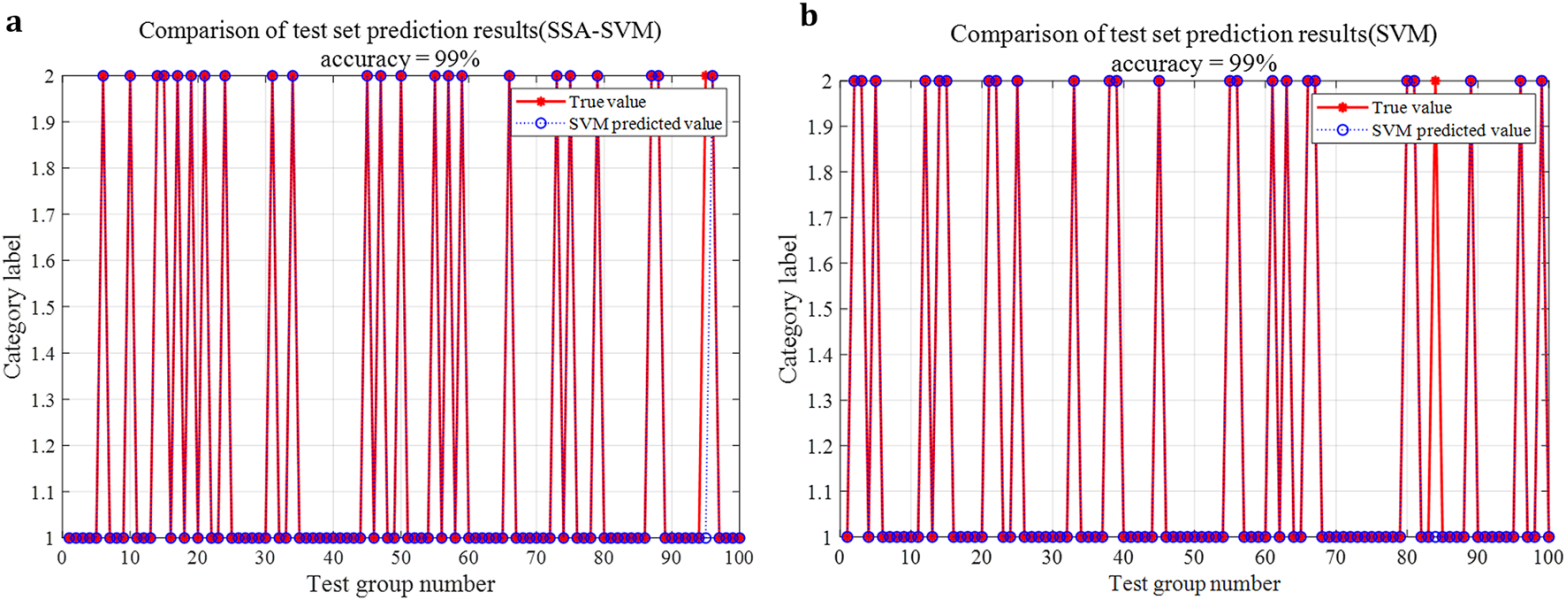
Fault diagnosis accuracy of SSA-SVM and CV-SVM. (**a**) Fault diagnosis accuracy of SSA-SVM. (**b**) Fault diagnosis accuracy of CV-SVM.

The penalty factor *C* and nuclear parameter *g* of SVM were optimized respectively by SSA and cross validation method, then the SSA-SVM model and CV-SVM model were used to diagnose the arc fault. The experimental results under the condition of 20A are shown in Fig. 14.

Ten repeated experiments were conducted under 15A, 20A and fluctuating working current conditions respectively to obtain the average value of fault diagnosis accuracy and diagnosis time. The diagnosis time in this research refers to the time spent from the moment when the upper computer collects the current signal until the arc fault is found. The experimental results are shown in Table 3.

**Table 3:** Comparison of fault diagnosis effects of different methods.

	15A	20A	Fluctuation	Diagnosis time/s
SSA-ELM	97.1%	99.1%	96.6%	36.249
GA-ELM	96.9%	98.4%	96.4%	42.359
PSO-ELM	96.7%	98.5%	96.4%	41.801
ELM	96.7%	97.5%	96.2%	33.162
SSA-SVM	97.7%	99%	95.2%	70.341
CV-SVM	96.3%	98%	95%	83.292

After using the proposed feature vector extraction method and SSA-ELM arc fault diagnosis model, at the cost of a small amount of diagnosis time, the detection accuracy is improved and the maximum average of the diagnosis accuracy can reach more than 99%. Compared with GA-ELM and PSO-ELM fault diagnosis models, SSA-ELM has faster convergence speed and higher accuracy.

The feature vectors extracted by the proposed method can also be used as the input of SVM. Under the condition of 20A current, the diagnosis accuracy of SVM can reach more than 98%, whatever the optimization method is SSA or cross-validation method. However, the optimization time of SSA is far less than that of cross-validation method.

When using SVM, the optimal penalty factor *C* and the kernel parameter *g* need to be found, while in ELM only the number of neurons in the hidden layer *L* needs to be optimized. So, through the optimization of SSA, although the fault diagnosis accuracy of the two is similar, ELM has huge advantages in the diagnosis time.

## Conclusion

The following conclusions are obtained by series arc fault experiments on typical three-phase loads:

1. An effective arc fault diagnosis model was proposed. VMD energy entropy, sample entropy of each cycle and the average value of half-cycle current after first-order difference were chosen as the input, and the extreme learning machine was optimized by sparrow search algorithm to accurately diagnose the arc fault.
2. Compared with ELM, GA-ELM and PSO-ELM, the SSA-ELM arc fault diagnosis model designed in this paper has faster optimization speed and higher diagnosis accuracy, it is more suitable for the diagnosis of arc fault. Compared with SSA-SVM and CV-SVM, SSA-ELM has great advantages in diagnosis speed.
3. SSA can also be applied to the optimization of SVM parameters and has a relatively fast optimization speed.
